# Supplementary figures and images for: Adaptations Accumulated under Prolonged Resource Exhaustion Are Highly Transient
Source: mSphere. 2020 Aug 12;5(4):e00388-20. doi: 10.1128/mSphere.00388-20 (PMC7426164; doi:10.1128/mSphere.00388-20)

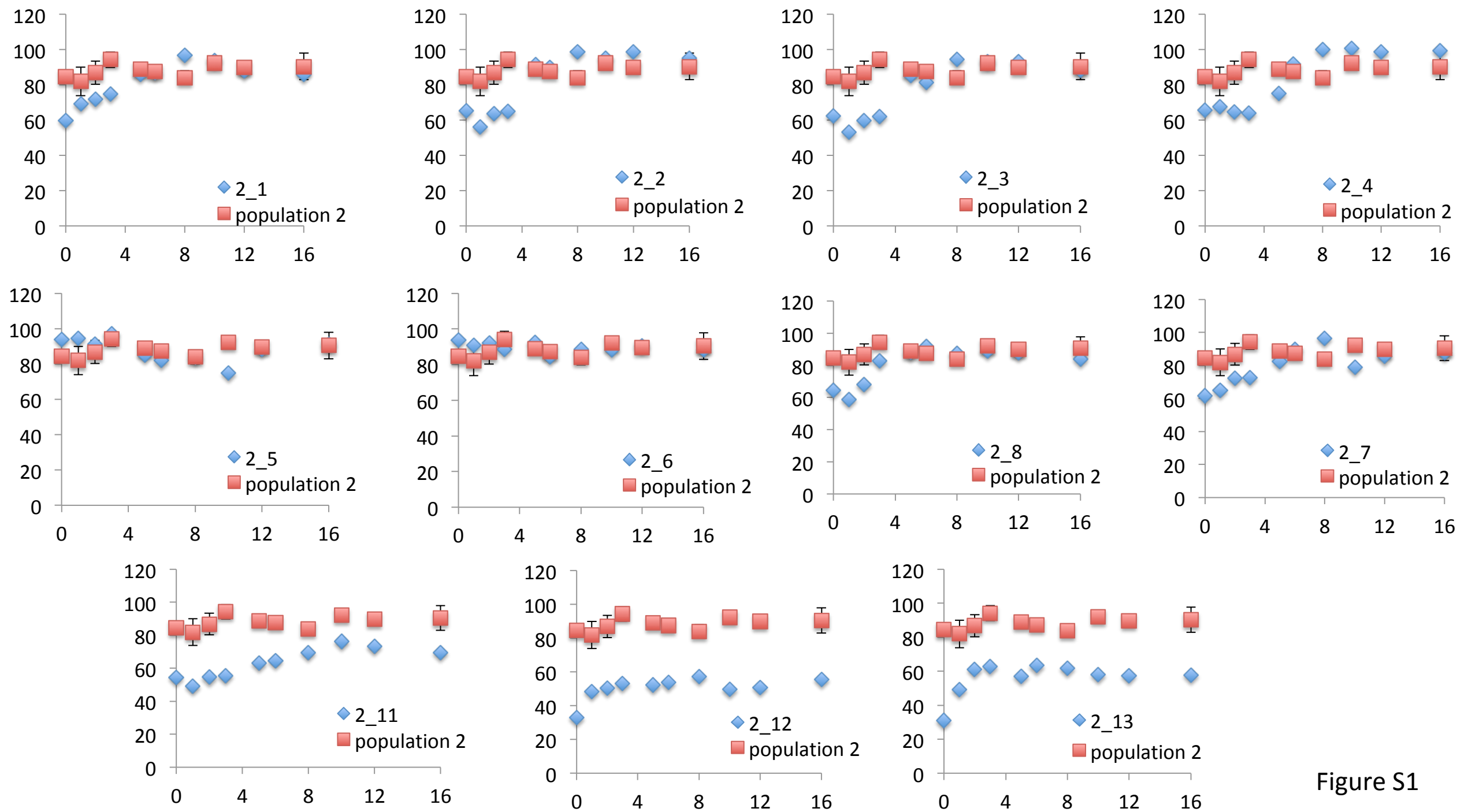

Figure S1

Supplement: FIG S1 [file mSphere.00388-20-sf001.pdf]

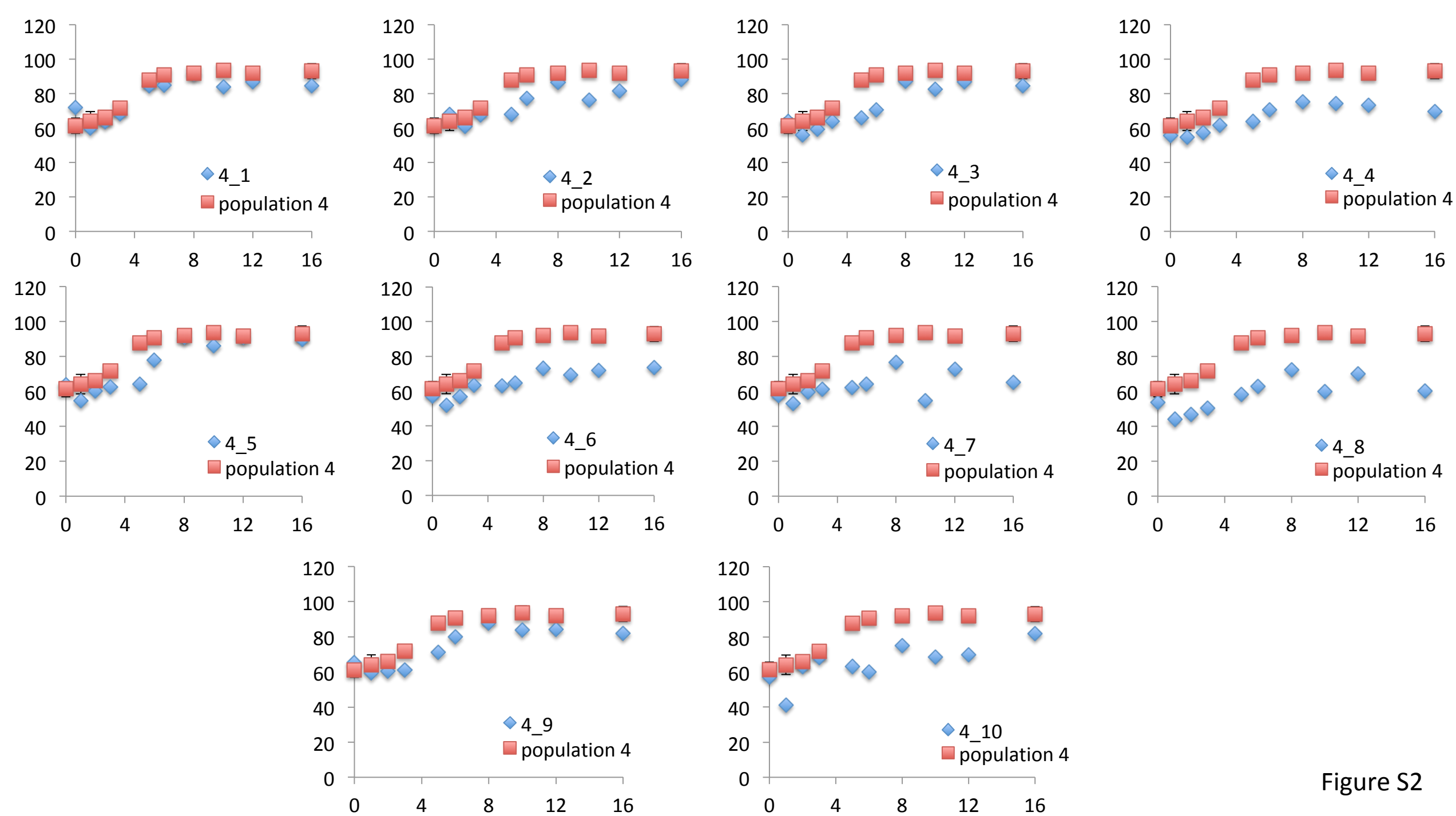

Figure S2

Supplement: FIG S2 [file mSphere.00388-20-sf002.pdf]
